# Recent findings on subjective well‐being and physical, psychiatric, and social comorbidities in individuals with schizophrenia: A literature review

**DOI:** 10.1002/npr2.12286

**Published:** 2022-08-02

**Authors:** Yupeng He, Ayako Tanaka, Taro Kishi, Yuanying Li, Masaaki Matsunaga, Shinichi Tanihara, Nakao Iwata, Atsuhiko Ota

**Affiliations:** ^1^ Department of Public Health Fujita Health University School of Medicine Toyoake Aichi Japan; ^2^ Department of Psychiatry Fujita Health University School of Medicine Toyoake Aichi Japan; ^3^ Department of Public Health Kurume University School of Medicine Kurume, Fukuoka Japan

**Keywords:** comorbidity, epidemiology, literature review, schizophrenia, subjective well‐being

## Abstract

**Aim:**

Care for people with schizophrenia is shifting the locus from long‐stay mental hospitals to nonspecialized community‐based settings. Knowledge on the care is not a sole property of psychiatric specialists. Community healthcare workers who do not specialize in psychiatry are recommended to learn more about schizophrenia. This review aimed to summarize recent findings on subjective well‐being and physical, psychiatric, and social comorbidities in individuals with schizophrenia.

**Methods:**

A literature review was conducted. We retrieved findings from existing systematic reviews and meta‐analyses as our preferred method. When data were not available, we referred to other types of studies.

**Results:**

As per our review, individuals with schizophrenia demonstrated poor subjective well‐being, happiness, and life satisfaction despite individual differences. Pharmacotherapy caused weight gain and constipation, whereas race and hospitalization might affect weight reduction. Individuals with schizophrenia demonstrated poor oral health, a high prevalence of noncommunicable diseases, and unique eating behaviors. Depression, sleep disorders, smoking, and alcohol and drug consumption were frequently found in the individuals. Research findings regarding problematic internet and smartphone use and stress perception were limited. Low health literacy and neglect of preventable behaviors were frequently seen in individuals with schizophrenia. They tended to be less educated, poor, unemployed, unmarried/unattached, and had poor social cognition, resulting in little social support and a small social network.

**Conclusion:**

Retrieving recent data, we confirmed that individuals with schizophrenia had poor subjective well‐being and suffer from various physical, psychiatric, and social comorbidities.

## 1 INTRODUCTION

Mental disorders have drawn much attention worldwide in recent years.^1^ Schizophrenia is a common mental disorder that affects more than 20 million people worldwide.^2^ A systematic review reported a median 12‐month and lifetime prevalence of 0.33% and 0.48%.^3^ Another systematic review demonstrated a median point, 12‐month, and lifetime prevalence as high as 0.39%, 0.40%, and 0.75%, respectively.^4^ In Japan, Okui estimated the point prevalence of schizophrenia, including schizotypal and delusional disorders, to be approximately 0.7% by using the national data of the Patient Survey.^5^ Japan has more than 3 00 000 psychiatric care beds.^6^ It is by far the biggest number among the member countries of the Organization for Economic Co‐operation and Development (OECD).^7^ About half of psychiatric inpatients suffer from schizophrenia or its allied disorders, and more than 70% of those are admitted for more than a year.^6^

The World Health Organization (WHO) advocates the deinstitutionalization of individuals with schizophrenia, that is, a shift of the locus of care for people with mental disorders from long‐stay mental hospitals to nonspecialized community‐based health settings to provide comprehensive, integrated, and responsive mental health and social care in the Comprehensive Mental Health Action Plan 2013–2030.^8^ In these circumstances, knowledge on the care is not a sole property of psychiatric specialists. Community healthcare workers who do not specialize in psychiatry are recommended to learn more about schizophrenia. Individuals with schizophrenia exhibit various symptoms, including positive (eg delusions, hallucinations) and negative (eg blunted affect, avolition) symptoms, which lead to physical, psychiatric, and social comorbidities.^9^, ^10^ This review summarizes relevant recent findings.

## 2 METHOD

In this review, we targeted the physical, psychiatric, and social comorbidities along with subjective well‐being, happiness, and life satisfaction. Overweight and obesity, oral health, noncommunicable diseases (NCDs), constipation, and eating behaviors were examined as physical comorbidities. Depression and sleep disorders; smoking, alcohol, and drug consumption; problematic internet and smartphone use; and stress perception and allostatic load were adopted as psychiatric comorbidities. Social comorbidities included health literacy and behaviors, socioeconomic status (such as education, employment, income, marital status, and family structure), and social cognitive bias, support, and network. Using these terms, potentially relevant papers were collected. We retrieved the findings from existing systematic reviews and meta‐analyses as our preferred method. When they were not available, we referred to existing cohort, case–control, and cross‐sectional studies. We searched the literature published up to February 2022 through PubMed. The existing research employed for this review was limited to clinical and epidemiological studies written in English.

## 3 RESULTS

We summarized the results in Table 1.

**TABLE 1:** Summary of findings

Subjective well‐being, happiness, and life satisfaction
Worsened subjective well‐being, happiness, and life satisfaction at a group level but varied by individual.
Overweight and obesity
High prevalence of obesity but varied by race.
Consequence of antipsychotics.
Oral health
Poor oral health: the greater number of missing and decayed teeth.
Noncommunicable diseases
High prevalence of chronic obstructive pulmonary disease, metabolic syndrome, type 2 diabetes, hypertension, and hypertriglyceridemia.
Constipation
Constipation and ileus caused by psychotropic medications, especially clozapine.
Eating behaviors
High dietary energy, sodium, and saturated fat.
Poor diet in fiber, fruit, and unsaturated fatty acids.
Depression and sleep disorders
High prevalence of comorbid major depressive disorder and sleep disturbances.
Smoking, alcohol, and drug consumption
High prevalence of smoking, drinking, and drug consumption.
Problematic internet and smartphone use
Problematic internet and smartphone use reported in South Korea.
Stress perception and allostatic load
Inconsistent evidence on whether to perceive more stress.
Related to greater allostatic load.
Health literacy and behaviors
Low health literacy and poor understanding of preventive behaviors.
Socioeconomic status: education, employment, income, marital status, and family structure
Low employment rate and income.
Less educated and likely to be unmarried/unattached.
Social cognitive bias, support, and network
Low ability to navigate social cues and behaviors.
Deficits in building relationships; lack of social support, community integration, and friends; and small size of social network.

### 3.1 Subjective well‐being, happiness, and life satisfaction

A Canadian cross‐sectional study by Fervaha et al^11^ revealed that young adults with schizophrenia demonstrated worse subjective well‐being and less happiness and life satisfaction than those without schizophrenia at a group level. Similar findings were confirmed in other studies on adults in Spain^12^ and United States.^13^ It was concurrently reported that a substantial number of individuals with schizophrenia felt high levels of subjective well‐being^11^ and happiness.^13^ Evidence for improvement of their subjective well‐being by pharmacological treatment and psychosocial therapy has been clarified.^14^, ^15^ Gutiérrez‐Rojas et al^12^ also reported that cognitive impairment might modulate the relationship between subjective happiness and functioning.

### 3.2 Physical comorbidities

#### 3.2.1 Overweight and obesity

A meta‐analysis reported that almost half of the individuals with schizophrenia were obese.^16^ Whether those with schizophrenia were overweight or obese might differ according to the treatment they were receiving. A review by Shah et al^17^ indicated that compared to healthy controls, individuals with schizophrenia who were antipsychotic‐naive and minimally treated showed lower body mass indices (BMIs) and no difference in waist circumferences. Antipsychotics would make those with schizophrenia overweight and obese. A review by Tarricone et al^18^ exhibited that weight and BMIs of antipsychotic‐naive patients increased after the beginning of antipsychotic medications. Another systematic review presented those antipsychotic medications, such as haloperidol, olanzapine, quetiapine, and risperidone, except for ziprasidone, were associated with weight gain and BMI increase in individuals with first‐episode psychosis.^19^ The longer the duration of antipsychotic medication, the higher the weight gain.^19^

Weight gain in individuals with schizophrenia may differ according to race. A meta‐analysis reported that Asian people presented lower weight gain than Western counterparts.^19^ A systematic review focused on underweight in individuals with schizophrenia and reported a high pooled prevalence of underweight of 17.6% in Japanese inpatients with schizophrenia, nearly 3‐fold higher than that in the patients worldwide.^20^

#### 3.2.2 Oral health

We found two meta‐analyses reporting poor oral health in individuals with schizophrenia.^21^, ^22^ In both of them, individuals with schizophrenia had higher decayed, missing, and filled teeth (DMFT) index scores. They had the greater number of missing and decayed teeth, but with fewer number of filled teeth, compared to healthy controls.

#### 3.2.3 Noncommunicable diseases (NCDs)

Individuals with schizophrenia were more likely to suffer from NCDs. A systematic review showed an about 1.5‐fold greater likelihood of suffering from comorbid chronic obstructive pulmonary disease in those with schizophrenia compared to the general population.^23^ Meta‐analyses reported a high prevalence of metabolic syndrome of more than 30% in those with schizophrenia.^16^, ^24^ Previous studies reported the prevalence of type 2 diabetes in individuals with schizophrenia, ranging between 8% and 23.3%.^25^ Some studies also reported the genetic predisposition for comorbidity of schizophrenia and type 2 diabetes.^26^, ^27^ A meta‐analysis indicated that about 19% of those with schizophrenia had hyperglycemia.^16^ It also showed that 38.7% and 39.3% of individuals with schizophrenia had hypertension and hypertriglyceridemia, respectively.^16^

#### 3.2.4 Constipation

Constipation often occurs in individuals with schizophrenia. The association between clozapine and constipation and ileus has been well examined. A meta‐analysis estimated that nearly one‐third of individuals with schizophrenia who were using clozapine experienced constipation.^28^ This study also reported that clozapine induced constipation more frequently than other antipsychotics. Another study showed that clozapine doubled the risk of ileus compared with other psychotropic medications.^29^ It caused fatal ileus more frequently than other psychotropic medications.

#### 3.2.5 Eating behaviors

We found two systematic reviews that focused on the eating behaviors of individuals with schizophrenia. One suggested that those with schizophrenia consumed higher dietary energy and sodium compared to healthy controls.^30^ Another revealed that, compared to healthy controls, those with schizophrenia were more likely to consume a poor diet in fiber, fruit, and unsaturated fatty acids and a diet rich in saturated fat.^31^

### 3.3 Psychiatric comorbidities

#### 3.3.1 Depression and sleep disorders

Depression is also prevalent in individuals with schizophrenia. A systematic review reported that a pooled estimate of the prevalence of the comorbid major depressive disorder was 32.6% in those with schizophrenia.^32^ It is suggested that even individuals with first‐episode schizophrenia indicated depressive symptoms more frequently than healthy controls.^33^ Existing findings were inconsistent regarding which of the two, patients with early‐ or chronic‐stage schizophrenia, expressed more severe depression.^33^ No significant difference in the rates of major depressive disorder was detected between patients with first‐episode schizophrenia and schizoaffective disorder.^34^

Sleep disturbances are often observed in individuals with schizophrenia. A systematic review reported that those with remitted schizophrenia showed a longer sleep duration, time in bed, and sleep latency than the healthy control.^35^ Another study found that insomnia (50%) and nightmare disorder (48%) were the most prevalent sleep problems among individuals with schizophrenia.^36^ Sleep disruption predicts the onset and persistence of psychotic experiences such as paranoia and hallucinations.

#### 3.3.2 Smoking, alcohol, and drug consumption

A multi‐institutional study in the United States revealed that smoking, drinking, and drug consumption were more prevalent in those with schizophrenia than in the general population.^37^ Smoking was suggested as a risk factor for schizophrenia incidence.^38^, ^39^

#### 3.3.3 Problematic internet and smartphone use

Problematic internet use, also called internet addiction, is characterized by persistent compulsive use of the internet that interferes with daily life.^40^ Lee et al^40^ reported that 22% of individuals with schizophrenia spectrum disorders suffered from problematic internet use in South Korea. They were more likely to have high levels of perceived stress and dysfunctional coping strategies.^40^ With the popularity of smartphones in recent years, problematic internet use has gradually turned into a form of problematic smartphone use.^41^ The South Korean researchers reported that the severity of problematic smartphone use was significantly associated with both high anxiety and low agreeableness.^41^ Since the subjects in these studies did not include healthy controls, it is unclear whether internet addiction is comparatively more frequent in those with schizophrenia.

#### 3.3.4 Stress perception and allostatic load

Stress has been linked to the etiology of schizophrenia because of its significant role in different stages of the illness.^42^ Gutiérrez‐Rojas et al^12^ did, and Nugent et al^42^ did not find that individuals with schizophrenia were more likely to perceive stress than healthy controls. Nugent et al focused on allostatic load, that is, the wear and tear of bodily experiences after responding to external stressors. They reported that those with schizophrenia had greater allostatic load compared to healthy controls, and greater allostatic load was found in both individuals with early course and chronic schizophrenia.^42^

### 3.4 Social comorbidities

#### 3.4.1 Health literacy and behaviors

A systematic review highlighted a low health literacy of individuals with schizophrenia.^43^ A cross‐sectional study reported that those with psychosis, 85% of whom suffered from schizophrenia, demonstrated a lack of understanding of preventive behaviors and poor knowledge of physical illnesses.^44^ Compared to the healthy control, they were less likely to undergo regular medical checkups and exercise, and to acknowledge the importance of early cancer detection and controlling NCDs.

#### 3.4.2 Socioeconomic status: education, employment, income, marital status, and family structure

Systematic reviews have indicated that individuals with schizophrenia tend to be less educated^45^ and exhibit a low employment rate^33^ than healthy controls. A Chinese study found that a lower income was associated with having schizophrenia at an individual level.^46^ Research using Danish population‐based data revealed that individuals with schizophrenia were likely to be unmarried/unattached.^47^, ^48^ An association between social dysfunction and marital status was found in individuals with schizophrenia in China.^49^ A Japanese study reported that approximately 10% of homeless people were diagnosed with schizophrenia or other psychotic disorders.^50^

#### 3.4.3 Social cognitive bias, support, and network

A meta‐analysis indicated that, compared to healthy controls, those with schizophrenia performed worse in social cognition, that is, a low ability to navigate social cues and behaviors inherently dependent on a knowledge base and set of skills.^51^ A psychological investigation revealed that social cognitive bias provided information about cognition, symptoms, and functioning related to interpersonal conflict in those with schizophrenia.^52^

It was highlighted that individuals with schizophrenia often had loneliness, deficits in building relationships, and lack of social support, community integration, and friends.^53^ A systematic review presented that a smaller social network size was associated with more severe psychiatric symptoms in individuals with schizophrenia.^54^ In an Australian nationwide survey, adults with psychotic illness (47% with schizophrenia and 16% with schizoaffective disorder) presented a high frequency of experiencing loneliness (80.1%) and a need for more friends (48.1%).^55^

A study in Taiwan reported a cross‐sectional association between a high level of social support, especially support from family, and symptomatic nonremission.^56^ A qualitative study in Pakistan suggested the association between social support and the willingness for treatment.^57^ With support from family, peers, and friends, they received positive emotional feelings, reduced depression, and gradually accepted regular medication and proper treatment. On the other hand, a systematic review pointed out that evidence for the effectiveness of peer support was insufficient.^58^

## 4 DISCUSSION

Retrieving recent data, mainly from systematic reviews and meta‐analyses, we have confirmed that individuals with schizophrenia suffer from poor subjective well‐being and various physical, psychiatric, and social comorbidities. Our review helps not only psychiatric specialists but also community healthcare workers who do not specialize in psychiatry learn more about the disorder and its management.

Individuals with schizophrenia had worse subjective well‐being and less happiness and life satisfaction than those without at the group level,^11^, ^12^ while the substantial heterogeneity among individuals with schizophrenia was appreciable as well.^11^, ^13^ This fact contributes to an elimination of the general misconception that all with schizophrenia are helpless. Given that cognition modulated the relationship between subjective happiness and functioning as reported,^12^ rehabilitation programs for cognitive impairment might improve recovery outcomes with a focus on subjective happiness in individuals with schizophrenia.^59^ Optimizing antipsychotic treatment, as well as psychosocial therapy, would improve subjective well‐being for individuals with schizophrenia.^14^, ^15^

For physical comorbidities, we targeted overweight and obesity, oral health, NCDs, constipation, and eating behaviors. Existing reviews indicate pharmacotherapy as a cause of weight gain^17^, ^18^, ^19^ and constipation.^28^, ^29^ Race^19^, ^20^ and hospitalization^20^ can also affect body weight. Individuals with schizophrenia tended to present a low DMFT index score,^21^, ^22^ suggesting poor health awareness and few opportunities for dental care, prevention, and treatment. This idea might be supported by a systematic review showing their low health literacy and academic achievement and neglect of preventable behaviors.^43^, ^44^, ^45^ Unique eating behaviors^30^, ^31^ and low health literacy and academic achievement^43^, ^44^, ^45^ could have contributed to the high prevalence of NCDs among the population.^16^, ^23^, ^24^, ^25^ Weight gain^16^, ^17^, ^18^, ^19^ and a high prevalence of smoking and drinking^37^ must also be monitored to prevent NCDs. Genetic influences may also account for comorbid diabetes.^26^, ^27^

For psychiatric comorbidities, we examined depression and sleep disorders, smoking, alcohol, and other drug consumption, problematic Internet and smartphone use, and stress perception and allostatic load. Depression and sleep disorders have long been known to be common psychiatric comorbidities. Our review confirms this finding.^32^, ^33^, ^34^, ^35^, ^36^ We found a clear indication of high prevalence of smoking, drinking, and drug consumption in the U.S. among individuals with schizophrenia.^37^ Problematic internet and smartphone use, an emerging addiction of the 21st century, was so far investigated only in South Korea.^40^, ^41^ Such findings should be duplicated in other countries. The existing findings were split regarding stress perception among individuals with schizophrenia.^12^, ^42^ More study findings are necessary to clarify this topic.

For social comorbidities, we explored health literacy and behaviors, socioeconomic status, and social cognitive bias, support, and network. As mentioned above, low health literacy and neglect of preventable behaviors were noted in individuals with schizophrenia.^43^, ^44^ This could contribute to a high prevalence of NCDs,^16^, ^23^, ^24^, ^25^, ^26^, ^27^ which are preventable to some extent. Being less educated,^45^ poor,^46^ unemployed,^33^ and unmarried/unattached^47^, ^48^, ^49^ were found as the features of individuals with schizophrenia. They also tend to perform worse in social cognition.^51^ As a result, they would have little social support and a small social network. This finding justifies the necessity of psychosocial treatment such as cognitive behavioral therapy, family interventions, social skills training, and supported employment.^60^

The strength of our review is that we summarized various fields of relevant studies in terms of subjective well‐being and physical, psychiatric, and social comorbidities. It is helpful for community healthcare workers who do not specialize in psychiatry. Systematic reviews and meta‐analyses were primarily included to ensure the findings are comprehensive and persuasive. We revealed under‐researched areas related to well‐being and comorbidities in individuals with schizophrenia and also provided indications for promising future research. A limitation of this study is that we did not conduct a systematic review with a meta‐analysis. Although we preferred recently published systematic reviews and meta‐analyses, our interpretation might have been biased by our subjective choices from the existing literature.

## AUTHOR CONTRIBUTIONS

AO conceived the study design; YH, TK, YL, MM, ST, and NI helped complete it. YH, AT, and AO collected and interpreted the references. YH, AT, and AO drafted the manuscript. The other authors revised the manuscript critically for important intellectual content. All authors have approved the final version of the manuscript for publication.

## Funding information

This study was supported by the Health and Labour Sciences Research Grant from the Ministry of Health, Labour and Welfare, Japan.

## CONFLICT OF INTEREST

The authors declare no conflict of interest.

## Data Availability

Data sharing is not applicable to this article as no new data were created or analyzed in this study.
